# Ethical considerations and management strategies for fertility preservation in women of reproductive age with malignant tumors: Chinese practices and perspectives

**DOI:** 10.3389/fendo.2026.1798693

**Published:** 2026-05-25

**Authors:** Ke Wang, Shihui Wang, Jianjiang Gao, Jiahuan Zhang, Jianqing Zhu

**Affiliations:** 1 Reproductive Medicine Center, Shanghai Key Laboratory of Maternal Fetal Medicine, Shanghai Institute of Maternal-Fetal Medicine and Gynecologic Oncology, Shanghai First Maternity and Infant Hospital, School of Medicine, Tongji University, Shanghai, China; 2 Department of Nursing, Fudan University Shanghai Cancer Center; Department of Oncology, Shanghai Medical College, Fudan University, Shanghai, China

**Keywords:** fertility management, fertility preservation, malignant tumor, reproductive ethics, women of childbearing age

## Abstract

**Background:**

Approximately 2 million women of childbearing age worldwide are diagnosed with malignant tumors each year, with tens of thousands of them facing the risk of fertility loss due to cancer treatment. With the advancement of tumor treatment technology, patient survival rates have significantly improved, and the demand for fertility preservation is increasing. However, tumor treatment damages ovarian function through various mechanisms, leading to temporary or permanent infertility. How to maximize the protection of fertility while controlling tumors has become a key issue in the overall management of tumors. Our review focuses on the ethical and regulatory landscape in China, while drawing selective international comparisons.

**Method:**

This article provides a comprehensive narrative review of the pathological mechanisms of fertility damage in women with malignant tumors of childbearing age, summarizes the effectiveness and safety of current major fertility preservation techniques (oocyte freezing, embryo freezing, ovarian tissue freezing, *in vitro* maturation techniques), analyzes the psychological challenges and family communication difficulties faced by patients in the decision-making process, explores the construction path of multidisciplinary collaboration models, and focuses on ethical and legal gaps in clinical practice.

**Result:**

① The survival rate of frozen mature oocytes after thawing can reach 80% -90%, with a cumulative live birth rate of about 33%. However, unmarried patients face legal gray areas such as cross institutional transportation of frozen eggs and the right to dispose of them after death; ② Embryo freezing technology is the most mature, and the pregnancy rate of frozen thawed embryo transfer is higher than that of fresh embryos, but it is only suitable for married patients, and their reproductive autonomy is limited after divorce; ③ Ovarian tissue freezing is suitable for patients in need of emergency treatment or pre puberty, with a sustained pregnancy/live birth rate of approximately 37.7% after transplantation, but there is a risk of tumor cell reintroduction (approximately 1%, with leukemia patients having the highest risk); ④ *In vitro* maturation technology does not require ovarian stimulation and does not delay treatment, but the live birth rate (8.9%) is significantly lower than that of mature egg freezing; ⑤ Patients generally have a need for fertility information (66% -100%), and anxiety and depression affect decision-making ability, requiring specialized and repetitive counseling and psychological intervention; ⑥ Multidisciplinary collaboration can optimize treatment timing, balance tumor control and fertility protection, but even if doctors have positive cognition, the actual referral rate is still not ideal; ⑦ Ethical challenges involve issues such as decision-making autonomy, the right to dispose of frozen gametes, long-term follow-up of offspring health, and social equity accessibility. However, there are divergent attitudes among the public towards the use of frozen gametes for research, emphasizing patients’ calls for inclusion in medical insurance and legal regulation.

**Conclusion:**

Fertility preservation requires the establishment of a patient-centered decision support system, incorporating psychological assessment and family communication into the counseling process; Establishing an institutionalized multidisciplinary collaboration model to eliminate the practical gap from cognition to referral; Improve ethical regulations and legal frameworks, clarify gray areas such as egg freezing, cross institutional transportation, and post death disposal rights for unmarried patients; Establish a long-term follow-up and social support network to accumulate offspring health and safety data. Only through multidimensional collaboration of technological application, ethical considerations, standardized management, and social support can we protect the fertility hope and dignity of cancer patients while curing the disease.

## 1 Introduction

Statistics indicate that approximately 2 million women of reproductive age are diagnosed with malignant tumors globally each year (1), among whom tens of thousands face the risk of fertility loss due to cancer treatment (2). Fertility is not only the foundation for human continuity and social development but also a crucial safeguard for the inheritance of civilization. From a sociological perspective, fertility generally refers to an individual’s potential ability to conceive and bear children under existing economic and social conditions (3).

The mechanisms by which malignant tumor treatments impair female fertility are complex and diverse. Chemotherapy drugs, particularly alkylating agents (such as cyclophosphamide), can lead to premature ovarian failure by inducing follicular apoptosis and depleting ovarian reserves (4). Pelvic radiotherapy directly irradiating the ovaries may cause oocyte DNA damage and even permanent loss of ovarian function (5). Surgical treatment for ovarian malignancies may necessitate the removal of partial or all ovarian tissues. Hematopoietic stem cell transplantation, as a core treatment for hematologic diseases, significantly improves survival rates but carries a 70% incidence of ovarian failure due to its conditioning regimens (chemotherapy and radiotherapy) (6). The resulting fertility decline from these treatments may push patients to meet the clinical diagnostic criteria for infertility. According to the International Classification of Diseases and Chinese Clinical Treatment Guidelines, infertility refers to the inability to achieve clinical pregnancy after 12 months of unprotected, regular sexual intercourse (7). For cancer patients, this condition may be either temporary or permanent.

With advances in cancer treatment, the survival rates of patients with malignant tumors have gradually improved (8). The demand for fertility preservation among young cancer patients is increasing year by year, encompassing not only physical rehabilitation but also the reconstruction of social roles and the restoration of family functions. Consequently, female fertility preservation has become a crucial component of comprehensive cancer management (9). Fertility preservation (FP) involves the use of surgical, pharmacological, or laboratory techniques to help patients retain their ability to have genetically related offspring when their fertility is at risk of decline or loss (10). The American Society of Clinical Oncology has repeatedly made recommendations on infertility in patients with malignant tumors after treatment, encouraging the exploration of the impact of cancer and treatment on fertility, and referring patients with fertility preservation intentions to reproductive medicine experts. The latest version of the 2025 guidelines further emphasizes the importance of full cycle fertility protection and individualized strategies (11). It is recommended to reassess fertility needs annually after treatment, when changing plans or preparing for pregnancy, and to include fertility counseling in the overall management of cancer.

In China, although the “Management Measures for Human Assisted Reproductive Technology” have been followed and the “Chinese Expert Consensus on Clinical Practice of Fertility Preservation in Women” has been introduced, with the increasing demand, reproductive medicine experts, patients, and their families still face many ethical dilemmas (12, 13). The ethical and social acceptance research on fertility preservation internationally provides important references for China. The study by Lewis et al. (14) showed that the public’s acceptance of frozen gametes for research purposes is not entirely consistent, suggesting that the issue of disposal rights must be clearly defined in informed consent; Czekajewska et al. (15) emphasized in their study of Polish patients that fertility preservation should be included in medical insurance and supported ethical and legal regulation of non-fertility; Louw é et al. (16) found in their survey in the Netherlands that interdisciplinary collaboration requires institutional safeguards.

Our article will elaborate on the mechanisms of damage to female fertility caused by malignant tumor treatment, protective technologies and strategies, interdisciplinary collaboration, and future development. The focus will be on analyzing the ethical challenges it faces and combining international experience to explore interdisciplinary management strategies and ethical regulatory recommendations that are in line with China’s national conditions, in order to provide theoretical and practical references for the protection of fertility in women with malignant tumors of childbearing age.

## 2 Review methodology

We conducted a comprehensive literature search in PubMed, CNKI, Wanfang Data, and Google Scholar for articles published up to December 2024. Search keywords included combinations of “fertility preservation”, “malignant tumor”, “oocyte cryopreservation”, “embryo cryopreservation”, “ovarian tissue cryopreservation”, “*in vitro* maturation”, “ethical considerations”, and “China”. We prioritized peer-reviewed original research, clinical guidelines, expert consensuses, and ethical analyses. Reference lists of included articles were also manually screened. Given the broad and integrative nature of this review, we did not apply a formal quality assessment or meta-analytic framework, which is consistent with a narrative review approach.

## 3 Mechanisms of fertility impairment in cancer patients

The impact of cancer and its treatment on fertility has become an important issue at the intersection of oncology and reproductive medicine. The damage mechanism is complex, involving the influence of the tumor itself as well as various iatrogenic factors such as radiotherapy, chemotherapy, surgical treatment, and hematopoietic stem cell transplantation (17). In addition, patient age is also an important influencing factor (18). Older patients have already experienced a decline in ovarian reserve function and reproductive cell quality, and the risk of fertility impairment further increases after receiving tumor treatment.

### 3.1 The impact of malignant tumors on fertility

Gene mutations related to malignant tumors can cause inflammatory factor release and metabolic disorders, which have a direct negative impact on oocyte, follicular development, and ovarian function. The germline mutation of breast cancer susceptibility gene (BRCA) in breast cancer patients has been confirmed to be related to accelerated follicular loss and early menopause (18). Tumor related stress, malnutrition, and ectopic hormone secretion can interfere with the normal function of the hypothalamus and pituitary gland, leading to abnormal secretion of gonadotropins (follicle stimulating hormone, luteinizing hormone), thereby causing anovulation or menstrual cycle disorders (such as amenorrhea and infrequent menstruation). Tumor cells have strong invasiveness and can infiltrate reproductive glands along the interstitium, directly damaging the microenvironment of primordial follicles, leading to hindered development and even death of primordial follicles. Meanwhile, tumor cells can also disrupt the pool of reproductive stem cells, further affecting ovarian function.

In addition to its direct impact on reproductive cells, certain tumors can also affect fertility by disrupting endocrine balance (19). Malignant tumors of the hematopoietic system may directly infiltrate the hypothalamic pituitary center, or cause dysfunction of endocrine glands such as the thyroid and adrenal glands, indirectly affecting the reproductive endocrine axis (20). And malignant tumors.

### 3.2 The impact of tumor treatment on fertility

Radiotherapy and chemotherapy are commonly used treatments for malignant tumors, but they can cause varying degrees of damage to ovarian function. The commonly used ionizing radiation in radiotherapy induces follicular apoptosis in a dose-dependent manner. Both oocytes and proliferating granulosa cells have high radiation sensitivity, and a dose of 2 Gy can cause 50% loss of follicles. When the cumulative dose reaches 14 Gy, it may lead to ovarian failure (21). Radiotherapy induced ovarian damage also includes damage to the stroma and vascular system, leading to ovarian tissue atrophy and fibrosis (21). Pelvic radiotherapy can cause damage to uterine blood vessels, leading to endometrial inflammation or muscle fibrosis, and affecting the development of the patient’s uterus (5).

Chemotherapy drugs block specific processes of tumor cell growth cycle through specific mechanisms of action, thereby inhibiting their survival and expansion (22). Among them, alkylating agents and platinum drugs have particularly significant damage to ovarian function. The gonadal toxicity of chemotherapy drugs depends on the type, dosage, administration method (oral or intravenous), and the combination of different chemotherapy drugs. The degree of damage caused is closely related to the patient’s age at the time of treatment and fertility before treatment. The use of alkylating chemotherapy drugs (such as cyclophosphamide) has a significant impact on ovarian reserve function and belongs to the category of high gonadal toxicity drugs (23). However, doxorubicin, epirubicin, carboplatin, and paclitaxel chemotherapy drugs belong to the category of moderately gonadotropic drugs (24).

At present, there is still limited evidence on the potential toxic effects of new cancer treatments (such as immune checkpoint inhibitors, antibody drug conjugates, small molecule targeted drugs, and monoclonal antibodies) on reproductive function, and there is an urgent need to conduct basic reproductive toxicity experiments and clinical studies related to new cancer treatment methods. In addition, myeloablative pretreatment before hematopoietic stem cell transplantation also has strong gonadal toxicity (6). The reproductive toxicity associated with these treatments not only affects the quality of life of patients but may also lead to long-term psychological and social problems (25).

## 4 Key technologies and strategies for fertility protection in unmarried women with malignant tumors of childbearing age

To provide a clear and comparative overview of the currently available options for fertility preservation in women of reproductive age with malignant tumors, **Table 1** summarizes four major techniques—oocyte cryopreservation, embryo cryopreservation, ovarian tissue cryopreservation, and *in vitro* maturation—across key dimensions including applicable populations, time requirements, success rates, oncological safety, ethical and legal gaps in China, as well as their respective advantages and disadvantages. The following subsections (2.1–2.4) elaborate on each technique in greater detail, with a focus on clinical efficacy, safety evidence, and unresolved challenges.

**Table 1:** Comparison of major fertility preservation techniques for women of reproductive age with malignant tumors.

Technique	Target population	Time required	Success rates (survival/pregnancy/live birth)	Risk of tumor recurrence	Ethical/legal gaps in China	Main advantages	Main disadvantages
Oocyte cryopreservation	Unmarried/married women; can delay treatment by 2–3 weeks; generally <40 years (may extend to 42 years)	Approximately 2–3 weeks (ovarian stimulation + oocyte retrieval)	– Post-thaw survival: 80%–90% – Cumulative live birth rate: ~33% (26–28)	Hormone-sensitive tumors (e.g., breast cancer): elevated estrogen from ovarian stimulation; potential recurrence risk requires further investigation (13)	– Unmarried patients: no clear regulation on cross-institutional transport – Posthumous disposition: legal gray area – Donation for research: public acceptance inconsistent (14)	Mature technology; applicable to unmarried patients; high survival rate	Requires treatment delay; hormonal stimulation may be unsuitable for hormone-sensitive tumors; success rate declines with age (<20% live birth for women >40 years (29, 30)
Embryo cryopreservation	Married women; can delay treatment by 2–3 weeks; generally <40 years (may extend to 42 years); not recommended if treatment cannot be delayed or for hormone-sensitive tumors (31)	Approximately 2–3 weeks (ovarian stimulation + oocyte retrieval + IVF + embryo culture)	– Post-thaw survival: >90% – Pregnancy/live birth rates after frozen-thawed embryo transfer higher than fresh embryo transfer (32) – Single blastocyst transfer reduces multiple pregnancy rate (33, 34)	Same as oocyte cryopreservation (risk from hormonal stimulation)	– Only applicable to married patients – After divorce: single mothers cannot undergo embryo transfer; reproductive autonomy limited – After patient’s death: ownership and disposition of embryos lack legal basis	Most mature technique; highest success rate; survival >90%	Only for married patients; requires treatment delay; hormonal stimulation; unusable after divorce
Ovarian tissue cryopreservation	Patients who cannot delay treatment; prepubertal girls; those requiring urgent therapy (35)	Can be performed immediately (no treatment delay); laparoscopic tissue retrieval	– Ongoing pregnancy/live birth rate: ~37.7% (36) – Live birth rate: orthotopic transplantation ~23% vs. heterotopic ~3% (37) – >150 healthy babies born worldwide by 2018 (38)	Risk of tumor cell reintroduction: ~1%; highest risk in leukemia patients (39, 40)	– Pathological examination before transplantation cannot completely exclude residual tumor cells – Informed consent must clearly state reintroduction risk	No treatment delay; applicable to prepubertal girls; can restore endocrine function	Risk of tumor reintroduction (~1%); requires laparoscopic surgery; relatively newer technique
*In vitro* maturation (IVM)	Patients requiring immediate treatment; pre-pubertal females; those unable to receive hormonal stimulation (41)	Can be performed immediately (no treatment delay); oocyte retrieval followed by 24–48 h *in vitro* culture	– *In vitro* maturation rate of immature oocytes: 60%–75% – Clinical pregnancy rate: 36.1% – Live birth rate: 25.9% – Post-thaw live birth rate: 8.9% (significantly lower than mature oocyte cryopreservation (42)	No hormonal stimulation → no recurrence risk for hormone-sensitive tumors; no tissue transplantation → no tumor reintroduction risk	– Significantly lower live birth rate than other techniques; patients must be fully informed – Long-term offspring health data insufficient	No treatment delay; no hormonal stimulation; no tumor reintroduction risk; applicable to pre-pubertal girls	Low live birth rate (especially post-thaw: 8.9%); suboptimal culture system; lower embryonic developmental potential (43–45)

All data in this table are derived from the references cited in the original manuscript or from explicit statements in the text. Success rates represent aggregated results from different studies; individual outcomes may vary.

### 4.1 The effectiveness and safety of cryopreservation of oocytes from unmarried women with tumors

Oocyte cryopreservation is primarily used for preserving surplus eggs during *in vitro* fertilization (IVF), for older women delaying childbearing due to social factors, and for patients requiring fertility preservation due to serious illnesses such as tumors. Influenced by differences in ethics and legal regulations, the eligible candidates and technical protocols for this procedure vary across countries and regions.

The technology is mainly divided into two categories: cryopreservation of immature oocytes and cryopreservation of mature oocytes. Immature oocytes can be retrieved at any time during the menstrual cycle and are suitable for cancer patients who cannot undergo ovarian stimulation or require immediate radiotherapy or chemotherapy (46). However, the *in vitro* culture system for immature oocytes is not yet fully developed, limiting its clinical application. Studies show that the *in vitro* maturation rate and fertilization rate of immature oocytes remain relatively low, with a clinical live birth rate of only 8.9%, significantly lower than that of mature oocyte cryopreservation (42, 47).

With advancements in embryo laboratory vitrification technology and intracytoplasmic sperm injection (ICSI), the live birth rate following IVF using cryopreserved mature oocytes has significantly improved. Data indicate that the survival rate of thawed mature oocytes can reach 80%–90%, with a cumulative live birth rate of approximately 33% (26–28). In 2013, the American Society for Reproductive Medicine (ASRM) issued the 《Clinical Guidelines for Mature Oocyte Cryopreservation》, formally recommending the integration of this technology into assisted reproductive clinical practice. To date, international literature has reported cases where female cancer patients successfully preserved fertility through mature oocyte cryopreservation and eventually achieved pregnancy and live birth using assisted reproductive technology (48).

Nonetheless, despite its applicability to unmarried patients with malignant tumors, the clinical implementation of mature oocyte cryopreservation continues to face several challenges in the following aspects:

① Limited clinical data. The application of this technology for fertility preservation in cancer patients has a relatively short history, and there is insufficient clinical data to confirm whether the malignancy itself affects oocyte quality.
② Unclear long−term safety for offspring. There remains a lack of long−term follow−up studies on children born from cryopreserved oocytes of cancer patients, and the impact on the health of subsequent generations has not been clearly established.
③ Age remains the most critical factor influencing the live−birth rate of oocyte cryopreservation. The relevant age refers to the woman’s age at the time of oocyte freezing, not her age at future embryo transfer. Goldman et al. (29) developed a predictive model indicating that to achieve a 75% live−birth rate, women aged 34, 37, and 42 would need to freeze approximately 10, 20, and 61 mature oocytes, respectively. The decline in oocyte quality associated with advancing age directly leads to reduced fertilization rates and increased aneuploidy. Studies show that the live−birth rate for cryopreserved oocytes in women over 40 is generally below 20%, while the number of oocytes required to be frozen increases substantially (30). This implies that older patients often need to undergo multiple egg−retrieval cycles, bearing greater physical, emotional, and financial burdens. Clinicians should provide individualized treatment recommendations based on the patient’s age and family financial situation.
④ Success rates and management of psychological expectations. The success rate of assisted reproduction using frozen oocytes may be lower for cancer patients compared to the non−cancer population. Therefore, physicians should fully inform patients of the risk of failure in subsequent fertility attempts before cryopreservation and provide appropriate psychological support.
⑤ Strict coordination of treatment timing. For patients whose anticancer therapy can be reasonably delayed, oocyte retrieval and freezing should be completed before radiotherapy or chemotherapy. Since ovarian stimulation to egg retrieval typically requires 2–3 weeks, close communication and precise coordination between oncologists and reproductive specialists are essential to establish an appropriate treatment sequence.
⑥ Potential risks for hormone−sensitive tumors. Ovarian stimulation leads to a significant rise in estrogen levels. Whether this increases the risk of recurrence or affects survival in patients with estrogen−sensitive tumors (e.g., breast cancer) requires further investigation (13).
⑦ Legal gaps in oocyte transportation. If the institution where oocytes were frozen before marriage differs from the institution where assisted reproduction is pursued after marriage, the inter−facility transfer of cryopreserved oocytes becomes an issue. Currently, China lacks clear laws or regulations governing such transfers.
⑧ Ethical and Legal Dilemmas Regarding Posthumous Disposition. The ownership of cryopreserved oocytes (e.g., whether parents can inherit them) and the permissible uses following the death of a cancer patient similarly fall into a gray area within existing legal and ethical frameworks. It is worth noting that even among the general population, the public’s acceptance of donating surplus eggs or embryos after *in vitro* fertilization for medical research is not entirely consistent (14). This suggests that for the more vulnerable group of cancer patients, clear and standardized informed consent must be obtained from patients regarding various possible future situations (including for scientific research) before implementing cryopreservation.

### 4.2 The effectiveness and safety of embryo cryopreservation in married women of reproductive age with malignant tumors

Embryo cryopreservation is currently the most established technique for female fertility preservation. This technology involves obtaining sperm and eggs from the couple or donors, performing fertilization *in vitro*, and cryopreserving the developed embryos—either at the cleavage stage (day 2–3) or the blastocyst stage (day 5–7)—in an ultra-low temperature environment (e.g., liquid nitrogen, -196 °C). The frozen embryos are thawed and transferred when the patient’s physical condition is suitable. This technique is primarily indicated for married women generally under 40 years of age, with at least two weeks available before planned pelvic radiotherapy or chemotherapy. If the patient has a strong desire for future childbearing and normal ovarian reserve, the age limit may be appropriately extended to under 42 years. However, it is not recommended for patients who cannot delay cancer treatment, whose general condition cannot tolerate ovarian stimulation and egg retrieval, or for whom specialist assessment suggests that procedures related to embryo manipulation might negatively impact tumor prognosis (31).

With the widespread adoption of vitrification technology, embryo freezing has become a common method for fertility preservation in infertile couples and cancer patients, characterized by relatively straightforward procedures and manageable costs. Studies indicate that compared to infertile women, cancer patients using frozen embryos for assisted reproduction show no significant differences in pregnancy rates, pregnancy-related risks, or delivery outcomes (49). Therefore, for eligible married cancer patients, embryo cryopreservation is considered a first-line strategy for fertility preservation within the field of assisted reproduction. Its advantages are primarily reflected in the high success rate of thawing and transfer. Research shows that compared to fresh embryo transfer, frozen-thawed embryo transfer yields higher pregnancy and live birth rates (32). Furthermore, compared to cleavage-stage embryo transfer, single blastocyst transfer not only further improves pregnancy and live birth efficiency but also effectively reduces the rates of multiple pregnancies and associated perinatal complications (33, 34).

Currently, the main methods of embryo cryopreservation are programmed (slow) freezing and vitrification (rapid) freezing. With the extensive application of vitrification, the survival rate of thawed embryos commonly exceeds 90%. It is important to note that this technique requires the use of exogenous hormones for ovarian stimulation prior to egg retrieval to obtain a sufficient number of eggs and embryos. Therefore, it is not suitable for patients requiring urgent treatment or those with estrogen-sensitive malignancies.

According to China’s 《Measures for the Administration of Human Assisted Reproductive Technology》, embryo cryopreservation is only applicable to married cancer patients. Before performing this procedure, clinicians should promptly inform patients of the following ethical and legal risks:

① It must be clearly explained to patients that the success rate of frozen embryo transfer is not 100%, and the pregnancy rate for cancer patients may be lower than that of the non-cancer population, meaning there is a possibility of assisted reproduction failure.
② After a successful transfer, some tumors (such as breast cancer) still carry a risk of recurrence during pregnancy. Termination of the pregnancy may be necessary if required by the patient’s medical condition.
③ If the patient divorces, current regulations do not permit single mothers to undergo frozen embryo transfer, which may result in the loss of their reproductive autonomy.
④ In the event of the patient’s death, there is no clear legal basis regarding the ownership and disposal of the preserved embryos. Therefore, thorough informed consent and contingency arrangements should be made prior to cryopreservation.

### 4.3 Efficacy and safety of ovarian tissue cryopreservation and transplantation in women of reproductive age with malignant tumors

Ovarian tissue cryopreservation has evolved from an experimental technique into a clinically recognized method for fertility preservation, particularly suitable for patients who cannot delay anticancer treatment and for prepubertal girls (35). This technique involves laparoscopically retrieving part of the ovarian cortex for cryopreservation. Once the patient’s condition is in remission, the tissue is thawed and transplanted back into the body (orthotopically or heterotopically). While slow freezing has been the traditional method, vitrification is increasingly used due to its simpler procedure, shorter time requirement, and theoretical advantage of reducing ice crystal damage. Studies indicate no significant difference between the two freezing methods in terms of primordial follicle survival rate, restoration of endocrine function post-transplantation, clinical pregnancy rate, or live birth rate (50, 51).

Clinically, most women of reproductive age hope to restore ovarian endocrine function and fertility by transplanting cryopreserved ovarian tissue after being cured of cancer. To promote the standardized application of this technology, China formulated the 《Chinese Expert Consensus on Ovarian Tissue Cryopreservation and Transplantation》 in 2018. Since Donnez et al. first reported the successful live birth of a healthy baby using this technique in 2004 (52), over 150 babies worldwide have been born following ovarian tissue cryopreservation and transplantation by 2018, with their health status showing no statistical difference compared to naturally conceived infants (38). Meta-analyses show that the ongoing pregnancy/live birth rate after ovarian tissue transplantation is approximately 37.7% (36). It is noteworthy that the transplantation site affects clinical outcomes: the live birth rate for orthotopic transplantation (e.g., onto the remaining ovary or near the original pelvic site) is significantly higher (about 23%) than for heterotopic transplantation (e.g., subcutaneous, about 3%) (37).

In China, Beijing Obstetrics and Gynecology Hospital, Capital Medical University, established the first ovarian tissue cryopreservation bank in 2012. In 2019, the first successful delivery in China following autologous transplantation of cryopreserved ovarian tissue via IVF technology was achieved. In 2020, the first case of natural pregnancy after transplantation led to a successful delivery in August 2021. These advancements mark significant breakthroughs in both theoretical and clinical practice in this field within China, offering new hope for fertility to young cancer patients.

However, ovarian tissue cryopreservation and transplantation still carry potential risks. Despite pathological examination before transplantation, a small number of tumor cells may remain in the tissue, posing a risk of tumor cell reintroduction and dissemination after transplantation. Studies estimate the probability of such an event to be approximately 1%, with the highest risk observed in leukemia patients (39, 40). Therefore, before performing cryopreservation, oncologists, reproductive specialists, and laboratory personnel must jointly assess the risks and develop an individualized plan in full communication with the patient and their family.

### 4.4 Efficacy and safety of *in vitro* maturation for women of reproductive age with malignant tumors

*In vitro* maturation of oocytes refers to the technique of culturing cumulus-oocyte complexes at the germinal vesicle stage to metaphase II maturity *in vitro*. With the advancement of tumor fertility, IVM has gained increasing attention due to its unique advantages in specific clinical scenarios and is no longer classified as an experimental technique by organizations such as the American Society for Reproductive Medicine (53). This technique requires minimal or no ovarian stimulation medication, can be performed at any time during the menstrual cycle without delaying cancer treatment, and thus is particularly suitable for patients needing immediate anticancer therapy. It also avoids the risk of tumor cell reintroduction associated with tissue transplantation (41). Furthermore, IVM can serve as an option for fertility preservation in female cancer patients who have not yet reached sexual maturity.

The core biological challenge faced by IVM is that the developmental potential of *in vitro* matured oocytes is generally lower than that of *in vivo* matured ones. This is primarily attributed to three issues: insufficient inherent developmental potential of the oocytes, susceptibility to spontaneous premature maturation after removal from the follicular microenvironment, and the lack of complete *in vivo* ovulatory cascade signaling (43–45). Oocyte maturation involves two key processes: nuclear maturation (resumption of meiosis) and cytoplasmic maturation (accumulation of key factors supporting fertilization and embryonic development) (54). A prospective randomized study showed that for immature oocytes from women with normal ovarian function, pretreatment with follicle-stimulating hormone alone did not significantly improve their *in vitro* maturation rate or clinical pregnancy rate. However, when FSH was combined with human chorionic gonadotropin, it synchronously enhanced both oocyte maturation rate and endometrial receptivity, leading to better clinical outcomes (55).

Nevertheless, whether the process involves maturing oocytes *in vitro* first and then freezing them or freezing immature oocytes first and then thawing and maturing them, the resulting embryos exhibit lower normal development rates and blastocyst formation rates compared to embryos derived from *in vivo* matured oocytes (31). This is likely because the *in vitro* culture environment struggles to fully replicate physiological conditions and is susceptible to various interfering factors. Data indicate that the *in vitro* maturation rate for immature oocytes is approximately 60%–75%, with clinical pregnancy and live birth rates of 36.1% and 25.9%, respectively. For oocytes that undergo cryopreservation after IVM, the post-thaw survival rate, clinical pregnancy rate, and live birth rate further decline to 59.8%, 10.7%, and 8.9%, respectively (42).

Currently, this technique still faces challenges such as inconsistent oocyte retrieval rates, limited maturation rates, and subsequent embryonic development rates. Future efforts will focus on improving aspiration needle technology, optimizing culture systems, and combining IVM with other fertility preservation methods like ovarian tissue cryopreservation to achieve more effective preservation of female fertility.

### 4.5 Impact of long−term cryopreservation on oocytes and embryos, and the health of offspring

With the widespread clinical application of oocyte and embryo cryopreservation technologies, they provide vital opportunities for fertility preservation in women who cannot conceive immediately. However, the potential impact of long-term cryopreservation on gametes and embryos remains a subject of concern in this field. The processes of freezing and thawing may induce alterations in cellular structures, such as affecting intracellular microtubule architecture and mitochondrial function, which could subsequently interfere with the early developmental potential of embryos. Studies indicate that prolonged cryopreservation might also accelerate the aging of certain subcellular components within oocytes, posing potential risks to their fertilization capacity and the subsequent normal development of embryos (56). Therefore, fully recognizing and evaluating these biological uncertainties is crucial during clinical decision-making.

Beyond developmental efficacy, the long-term health of offspring born from cryopreserved gametes or embryos is another key focus. Most current research suggests no significant difference in birth defects or growth and development between offspring conceived from frozen eggs/embryos and those from fresh cycles (57). Nonetheless, given the relatively limited timeframe of large-scale application of these technologies, their potential long-term effects on offspring health still require further validation through large-scale, long-term follow-up studies.

Consequently, when providing fertility preservation counseling, physicians and medical institutions have the responsibility to fully and objectively explain the potential risks and uncertainties associated with long-term cryopreservation to patients, and to emphasize the importance of long-term follow-up. On this basis, through thorough informed consent and shared medical decision-making, patients can be assisted in making choices that align with their personal circumstances and value judgments.

### 4.6 Other fertility preservation technologies

Technologies such as *in vitro* culture of primordial and preantral follicles, artificial ovaries, and the differentiation of induced pluripotent stem cells into germ cells (58) are currently still in the laboratory research stage. The *in vitro* culture of primordial and preantral follicles is a potential fertility preservation method that allows immature follicles to be extracted from a patient’s ovaries and cultured in an *in vitro* environment. The advantage of this approach is that it avoids hormonal stimulation of the patient, thereby reducing potential health risks. Furthermore, it offers a rapid and effective fertility preservation option for patients who need to commence cancer treatment promptly.

Artificial ovaries represent another innovative method, constructing a system that mimics the natural ovarian environment through the combination of biomaterials and germ cells. The goal of this technology is to provide patients with an alternative ovarian environment capable of producing mature oocytes, which is particularly valuable for patients who have lost natural ovarian function due to medical treatment.

The technology of differentiating induced pluripotent stem cells (iPSCs) into germ cells is a highly promising area of research. By reprogramming adult cells into stem cells and then inducing their differentiation into germ cells, this method holds the potential to offer new hope to patients who cannot preserve fertility through conventional means—for example, those unable to produce germ cells due to certain genetic conditions.

Although these technologies remain in the research stage, their development brings new hope and possibilities to the field of fertility preservation. As research deepens and technologies mature, these approaches may provide more fertility options for young cancer patients, while simultaneously sparking new discussions concerning ethical, legal, and social acceptance issues.

## 5 Management strategies for fertility protection in unmarried women with malignant tumors of childbearing age

### 5.1 Patient/family counseling and decision-making services

A systematic review shows that 66% to 100% of cancer patients indicate a need for fertility information, especially for those of childbearing age who have no children but still have plans to have children (55). The decision to preserve fertility is not only a medical choice for cancer patients and their families, but also an emotional and ethical challenge. Diversified consulting services and precise decision support tools have emerged, which can create a diversified decision support system for patients, reduce decision-making errors caused by information shortages and cognitive biases, and thus reduce decision-making conflicts and regrets. In addition, patients often experience anxiety and depression after diagnosis, and their ability to receive and understand fertility information is limited. This requires the counseling process to be professional, repetitive, and individualized. The ideal fertility preservation consultation should be integrated throughout the entire process of tumor treatment. The initial consultation should be conducted as soon as possible after diagnosis and before treatment, including: ① the potential impact of tumor treatment plans on fertility; ② Optional fertility preservation techniques and their success rates, risks, and time windows; ③ Economic costs and medical insurance policies; ④ The issue of future disposal rights for frozen gametes/embryos. Subsequent consultations should reassess fertility needs and physical condition after treatment is completed and before preparing for pregnancy.

### 5.2 Psychological intervention and social support

Women of childbearing age with tumors often face the inner conflict of “survival first or fertility first”. Some patients give up fertility preservation due to concerns about delayed treatment or increased risk of recurrence, resulting in long-term psychological regret. Psychological intervention should include: ① individual psychological counseling to help patients cope with the dual pressures of illness and childbirth; ② Couple/family counseling to promote family members’ understanding and support for reproductive decisions; ③ Peer support, sharing experiences through patient groups to alleviate feelings of isolation.

For married patients, the spouse’s attitude directly affects fertility preservation decisions; For unmarried patients, parents may make decisions on their behalf, involving the issue of proxy consent rights for minors or adult unmarried patients. In clinical practice, patients’ autonomous wishes should be respected, and family members should be guided to become supportive resources rather than decision makers.

### 5.3 Exploration and practice of multidisciplinary collaborative diagnosis and treatment model

For female cancer patients with fertility needs, clinical decision-making and implementation of fertility preservation should be integrated throughout the entire treatment process. The preservation of fertility in cancer patients is a systematic project involving multiple disciplines such as oncology, reproductive medicine, psychology, and genetics, and its diagnosis and treatment decisions directly affect the quality of life and survival of patients. The multidisciplinary diagnosis and treatment model provides patients with a full cycle, personalized diagnosis and treatment plan by building an interdisciplinary collaboration system. Balancing tumor control with protection of reproductive potential. This model not only optimizes treatment timing, improves prognosis, enhances patient compliance and psychological adaptation, but also helps to make rational use of medical resources. The 2020 guidelines of the European Society for Human Reproduction and Embryology also emphasize the importance of an effective management model centered on teamwork, service integration, and dedicated coordination (42). However, challenges still exist in reality. Louwé et al. (16) found in a survey of oncologists in the Netherlands that although doctors generally recognize the importance of fertility protection, actual clinical referral and communication practices are far from ideal. This fully demonstrates that establishing a standardized interdisciplinary collaboration model with institutional safeguards and dedicated coordinators is crucial for bridging the gap from “cognition” to “action”.

The value of MDT mode mainly lies in three aspects: firstly, introducing fertility preservation assessment in the early stage of tumor diagnosis to avoid missing the best preservation opportunity; Secondly, individualized treatment plans should be developed based on the specific situation of the patient, balancing the dual needs of tumor treatment and fertility protection; The third is to provide comprehensive health management, including post treatment fertility guidance and psychological support.

## 6 Ethical and legal considerations

To provide a structured ethical analysis, we adopt the Princip list framework proposed by Beauchamp and Childress, which organizes ethical issues in clinical medicine around four core principles: respect for autonomy, beneficence, non-maleficence, and justice. Each of the following subsection’s maps identified challenges onto one or more of these principles.

① Respect for autonomy: informed consent and decision-making capacity: Patients need to make fertility preservation decisions within a short period of time after diagnosis, which may be affected by anxiety and insufficient information. Patients need to make fertility preservation decisions within a short period of time after diagnosis, which may be affected by anxiety and insufficient information. A systematic review shows that 66% to 100% of cancer patients indicate a need for fertility information, especially those of childbearing age who have no children but still have plans to have children (55). The ideal fertility preservation consultation should be integrated throughout the entire process of tumor treatment, including: ① the potential impact of tumor treatment plans on fertility; ② optional fertility preservation techniques and their success rates, risks, and time windows; ③ economic costs and medical insurance policies; ④ the issue of future disposal rights for frozen gametes/embryos.

However, patients often experience anxiety and depression after diagnosis, and their ability to receive and understand fertility information is limited. This requires the counseling process to be professional, repetitive, and individualized. Doctors should provide sufficient information, avoid excessive guidance, and respect the patient’s final decision.

Principle−based reflection (autonomy): Respect for autonomy requires more than simply obtaining a signature on a consent form. It demands that the patient be given sufficient time (when medically feasible), understandable information, and psychological support to make a voluntary choice. In the context of fertility preservation, autonomy is further challenged by the patient’s acute cancer diagnosis and the urgency of treatment decisions. Therefore, repeated counseling sessions, decision aids, and the involvement of fertility navigators are not optional but ethically necessary to safeguard autonomous decision−making.

② Beneficence vs. non-maleficence: balancing fertility preservation with oncological safety:

From beneficence (doing good): Fertility preservation offers cancer patients the possibility of having genetically related offspring after cure, which significantly improves long−term quality of life and psychological recovery. However, the success rates are not 100%, and older patients often need to undergo multiple egg−retrieval cycles, bearing greater physical, emotional, and financial burdens (29, 30).

From non−maleficence (avoiding harm): Ovarian stimulation leads to a significant rise in estrogen levels. Whether this increases the risk of recurrence or affects survival in patients with estrogen−sensitive tumors (e.g., breast cancer) requires further investigation (13). Ovarian tissue cryopreservation carries a risk of tumor cell reintroduction after transplantation, estimated at approximately 1%, with the highest risk observed in leukemia patients (39, 40). The tumor itself has a risk of recurrence, especially malignant tumors such as breast cancer may recur during pregnancy, leading to the need to terminate pregnancy. Existing research shows that there is no significant difference in the health status of offspring born from frozen eggs compared to natural pregnancies, but long−term follow−up data is still limited. Patients should be informed of this uncertainty.

Principle−based reflection (beneficence & non−maleficence): The ethical tension between beneficence and non−maleficence lies at the heart of fertility preservation. Clinicians must weigh the potential future benefit of genetic parenthood against immediate risks—including delayed cancer treatment, hormone exposure, and the rare but catastrophic risk of reintroducing malignant cells. This balancing act is highly individual: for a patient with a slow−growing, hormone−insensitive tumor, beneficence may tip the scale toward aggressive fertility preservation; for a patient with aggressive leukemia requiring immediate myeloablative therapy, non−maleficence may argue against any intervention that postpones treatment. A shared decision−making model that transparently communicates both benefit and harm probabilities is essential to operationalize these principles.

③ Justice: social equity and accessibility:

Egg freezing costs are relatively high, and most regions are not covered by medical insurance, which may result in poor patients being unable to access fertility preservation opportunities. The study by Czekajewska et al. (15) shows that patients generally call for the inclusion of fertility preservation in medical insurance, which provides a reference for policymaking in China.

In addition, the age−dependent success rate of oocyte cryopreservation means that older patients need to freeze more oocytes and undergo more cycles, imposing a greater financial burden. This creates a double inequity: older patients already have lower success rates, yet they must pay more to achieve a modest chance of live birth.

Principle−based reflection (justice): The principle of justice demands that fertility preservation not become a privilege reserved for the wealthy. Currently, the out−of−pocket cost of oocyte or embryo cryopreservation in China (approximately ¥20,000–50,000 per cycle, plus annual storage fees) places it beyond the reach of many young cancer patients, especially those from rural areas or with lower socioeconomic status. This raises a fundamental question: should fertility preservation be considered an integral part of oncology care, analogous to pain management or psychological support? Drawing on international examples—such as the inclusion of fertility preservation in national health insurance in Israel and some European countries—China may consider pilot programs or charitable foundations to reduce economic barriers. Without such measures, the promise of fertility preservation will remain unequally distributed.

④ Extended non−maleficence and autonomy: posthumous disposition, cross−institutional transport, and reproductive rights of unmarried women.

for unmarried patients: If the institution where oocytes were frozen before marriage differs from the institution where assisted reproduction is pursued after marriage, the inter−facility transfer of cryopreserved oocytes becomes an issue. Currently, China lacks clear laws or regulations governing such transfers. The ownership of cryopreserved oocytes (e.g., whether parents can inherit them) and the permissible uses following the death of a cancer patient similarly fall into a gray area within existing legal and ethical frameworks.

For married patients: If the patient divorces, current regulations do not permit single mothers to undergo frozen embryo transfer, which may result in the loss of their reproductive autonomy. In the event of the patient’s death, there is no clear legal basis regarding the ownership and disposal of the preserved embryos. General ethical concern: Even among the general population, the public’s acceptance of donating surplus eggs or embryos after *in vitro* fertilization for medical research is not entirely consistent (14). This suggests that for the more vulnerable group of cancer patients, clear and standardized informed consent must be obtained from patients regarding various possible future situations (including for scientific research) before implementing cryopreservation.

Principle−based reflection (autonomy + non−maleficence): The disposition of frozen gametes and embryos after a patient’s death or divorce represents a clash between respect for autonomy and the principle of non−maleficence (preventing harm to the patient’s family or to society’s moral sensibilities). From an autonomy perspective, a patient’s prior written directive—specifying whether gametes should be discarded, donated to research, or inherited by parents—should be legally binding. However, current Chinese regulations do not provide such clarity, leaving patients and their families in a vulnerable position. Non−maleficence also applies here: without clear legal rules, families may face prolonged disputes or emotional harm. Therefore, we recommend that fertility centers implement a mandatory, legally reviewed “advance directive for gamete/embryo disposition” as part of the informed consent process, covering scenarios of death, divorce, loss of capacity, and long−term loss of contact. Furthermore, the restriction on embryo transfer for single women after divorce warrants re−examination from the standpoint of reproductive autonomy, as it effectively negates the patient’s prior autonomous choice made during marriage.

## 7 International perspectives from the literature

While China has made notable progress in fertility preservation technologies, its ethical and regulatory framework remains distinct from those in Western countries and other Asian regions; **Table 2** provides a comparative overview of these differences.

**Table 2:** International comparison of selected ethical and policy aspects related to fertility preservation in female cancer patients.

Country/Region	Applicable population/marital restrictions	Insurance coverage	Regulatory/legal characteristics	Clinical practice/referral features	Key ethical issues highlighted
China (12, 13)	– Unmarried women: permitted for oocyte cryopreservation – Embryo cryopreservation: only for married couples – Divorced women: cannot use frozen embryos	Generally not covered (out−of−pocket)	– Cross−institutional transport of frozen oocytes: no clear regulation – Posthumous disposition: legal gray area – Ownership of frozen gametes/embryos after death: undefined	– Multidisciplinary collaboration exists but referral gaps persist – Hormone−sensitive tumors (e.g., breast cancer): concerns about ovarian stimulation safety	– Decision−making autonomy under time pressure – Informed consent completeness – Reproductive rights of unmarried/divorced women
United States (11, 14)	– Not specified in cited references – ASCO guidelines apply to all cancer patients	Not mentioned in cited references	– No specific regulation mentioned – Public attitudes toward donating frozen gametes for research: not uniform	– ASCO 2025 guidelines recommend full−cycle fertility protection and individualized strategies – Recommend annual reassessment of fertility needs	– Need for clear informed consent regarding disposal rights for research use
Netherlands (16)	Not specified	Not mentioned	No specific regulation mentioned	– Oncologists: recognize importance of FP – Actual referral and communication: far from ideal – Need: institutional safeguards and dedicated coordinators	– Gap between “cognition” and “action” – Need for systematic MDT implementation
Poland (15)	Not specified	Not covered; patients strongly advocate for inclusion in health insurance	Patients support ethical and legal regulation of non−fertility use of reproductive cells	Not mentioned	– Social equity/justice – Patient advocacy for policy change
United Kingdom (14)	Not specified	Not mentioned	No specific regulation mentioned	Not mentioned	– Public views on donating biological samples (including frozen gametes) for research: inconsistent – Implication: disposal rights must be clearly defined in informed consent

“Not mentioned” indicates that the cited reference(s) for that country/region did not provide information on that specific dimension. This table strictly adheres to the content of the references cited in the present review; no extrapolation has been made.

As shown in **Table 2**, China is distinct in its explicit marital restrictions for embryo cryopreservation and regulatory gaps for unmarried women. Other countries highlight different priorities: the US focuses on guideline−driven individualized care, the Netherlands on implementation barriers, Poland on insurance advocacy, and the UK on public attitudes and systemic coordination. These international perspectives may inform future policy and practice improvements in China.

## 8 Conclusion

With significant advances in cancer treatment, the survival rates and prognosis for young women with malignant tumors have continuously improved, making post-treatment fertility needs increasingly prominent. In this context, fertility preservation has evolved from an emerging technology into an integral component of comprehensive cancer care and reproductive health management. It concerns not only the possibility of biological continuation for patients but also profoundly impacts their long-term quality of life, psychological recovery, and social identity.

However, the clinical implementation of fertility preservation faces a series of intertwined ethical, medical, and social challenges. On the technical front, strategies such as oocyte cryopreservation, embryo preservation, ovarian tissue transplantation, and *in vitro* maturation of oocytes each have specific clinical indications, limitations in efficacy, and potential risks, requiring strict adherence to individualized principles. At the ethical and legal levels, complex issues arise, including ensuring patient autonomy and informed consent, balancing treatment timing with oncological safety, determining the long-term ownership and disposal of gametes and embryos, addressing the inheritance rights concerning posthumous embryos, safeguarding the reproductive rights of unmarried women, and disclosing and following up on long-term health risks to offspring. These dilemmas necessitate that clinical decision-making transcends mere technical feasibility, delving into multiple dimensions such as the dignity of life, family relationships, and social justice. To address these challenges, we propose the following systematic ethical and management strategy recommendations:

① Patient centered decision support system: Establish a fertility preservation counseling process that runs through the entire process of tumor diagnosis and treatment, integrate psychological assessment and family communication, and ensure that patients make independent decisions in a fully informed and emotionally stable state.
② Institutionalized multidisciplinary collaboration model: incorporating professionals from oncology, reproductive medicine, psychology, nursing, ethics and law into a fixed collaborative team, clarifying responsibilities and workflow, and eliminating the practical gap from “cognition” to “referral”.
③ Improve ethical regulations and legal frameworks: Based on existing regulations, develop specialized guidance or industry standards for gray areas such as egg cryopreservation, cross institutional transportation, and post death gamete disposal for unmarried patients, clarifying the rights and responsibilities of all parties involved. Meanwhile, drawing on international experience (15), explore the feasibility of incorporating fertility preservation into medical insurance to promote social equity.
④ Building a long-term follow-up and social support network: establishing a regular confirmation mechanism for frozen gametes/embryos, respecting the dynamic changes in patients’ wishes; Conduct long-term follow-up studies on offspring health and accumulate safety data; Reduce the economic and psychological burden of patients through public welfare funds, psychological assistance, and other forms.

Fertility protection for cancer patients of childbearing age is not only a systematic project related to quality of life and continuity, but also an important manifestation of modern medical humanistic care and technological integration. Clinical doctors should proactively provide fertility protection related consultations in the early stages of diagnosis and treatment, and develop personalized fertility protection plans for patients. Considering that fertility protection has strong professional attributes, it is recommended to achieve fertility protection through interdisciplinary collaboration. Only through the multidimensional collaboration of prudent technological application, in−depth ethical consideration, standardized management, and systemic social support can we truly safeguard their hope for fertility and dignity of life while curing the disease.
